# Supplementary material for: Object Detection of Small Insects in Time-Lapse Camera Recordings
Source: Sensors (Basel). 2023 Aug 18;23(16):7242. doi: 10.3390/s23167242 (PMC10459366; doi:10.3390/s23167242)
Supplement: Supplementary file 1 [file sensors-23-07242-s001.zip › sensors-2533987-SI.pdf]

Supplementary Materials: Object Detection of Small Insects in Time-Lapse Camera Recordings

Kim Bjerge , Carsten Eie Frigaard and Henrik Karstoft

1. Introduction

This document contains supplementary material to the journal article "Object Detection of Small Insects in Time-Lapse Camera Recordings". As such, the content of this document should only be viewed in the context of the main article.

The supplementary material provides additional examples of images with labeled insects extracted from the dataset. It also illustrates the effects of motion-informed enhancement using images with labeled insects and the effectiveness of model detection in test images. Insects, shown in this material, were detected by the best trained YOLOv5 model with motion-informed enhancement.

1.1. Annotations and detections

Figure S1 shows examples of time-lapse images from camera locations S2-1 and S4-0. Three honeybees are marked with green bounding boxes to show model detections (true positive) of the small insects on a large background of plants. Two honeybees are marked with blue bounding boxes to show annotations for which the model did not detect (false positive).

Figure S2 shows more examples of cropped images of labels and model detections. Detections of honeybees are shown from two different camera locations (S1-1 and S4-4). False positive detections (red labels) were typically detected on either flower buds or background areas in the transition between flower and plant. False negative detections (blue labels) were in many situations due to partly visible honeybees occluded by flowers or plants. Some of the false negative detections in Figure S2 could not be visibly explained. However, small blurred bees were difficult to detect.

1.2. Motion-Informed Enhanced images

Figure S4 shows an example of motion-enhanced images created from a time-lapse recording with an interval of 30 seconds. Even though the images were recorded in a greenhouse with no wind, the plants did move, as seen from the red shadows surrounding the plants. Four honeybees are visually present (yellow labels) in the images enhanced with red color, showing that the bees move. Figure S3 shows the effect of motion-enhanced images with moving insects.

Figure S5 shows that the YOLOv5 model trained with motion-enhanced images detects more insects than that trained with only RGB images. In week 27 from camera S4-0, 619, or an increase of approximately 59%, more insects were detected for a YOLOv5 model trained with motion-enhanced images.

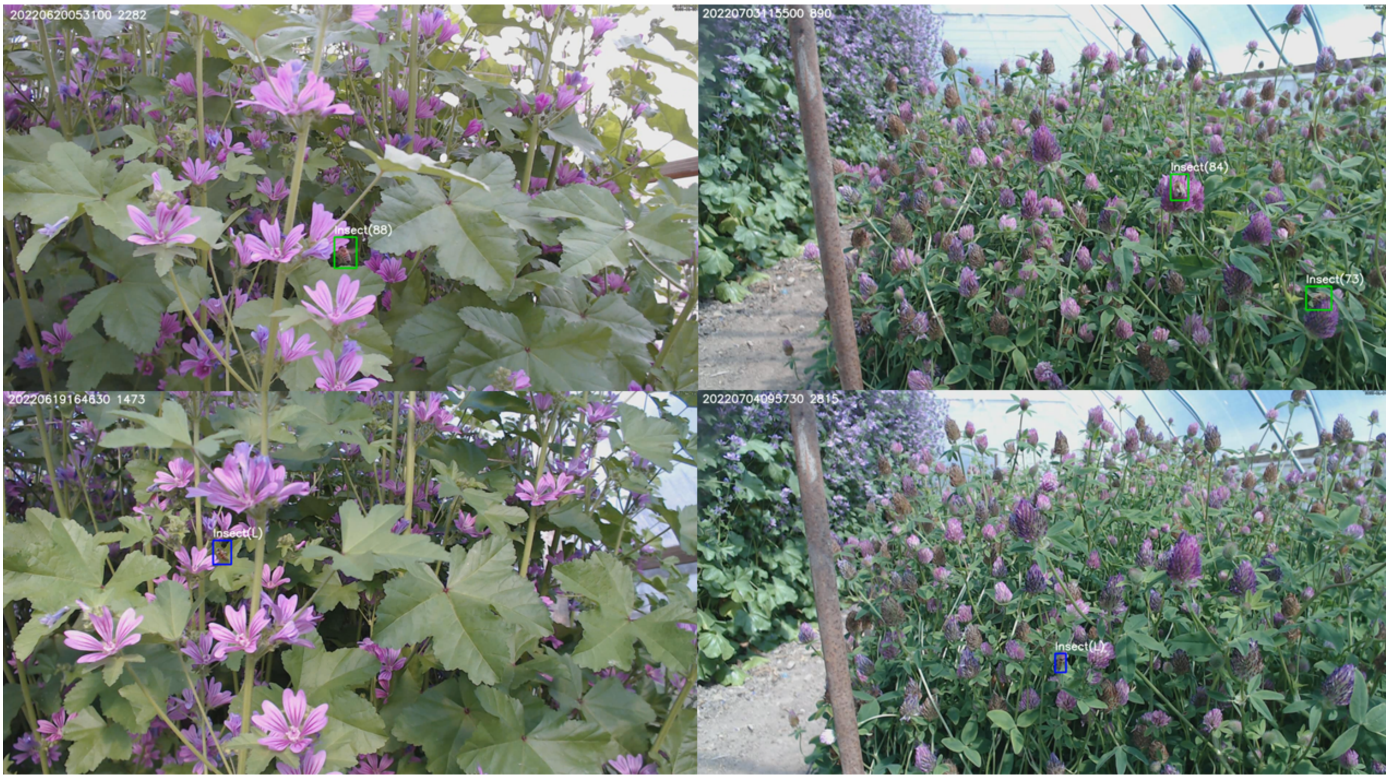

Figure S1. Example of time-lapse test images from camera S2-1 (left) and S4-0 (right). True positive (TP) detections and labels are marked with green, and labels (marked with 'Insect(L)') with false negative (FN) detections are marked with blue. The numbers are the confidence score from YOLOv5.

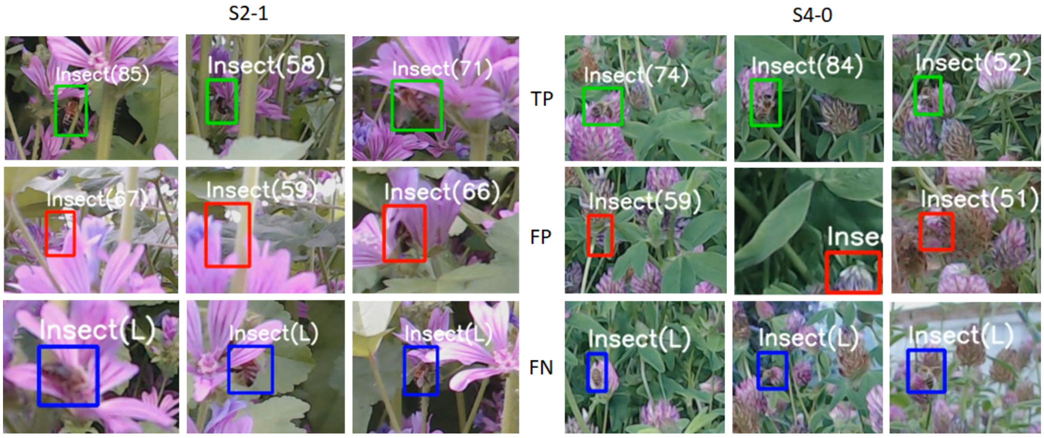

Figure S2. Example of labels and detections in the test dataset. True positive (TP) detections are marked with green. False positive (FP) detections are marked with red. False negative (FN) detections are marked with blue. The numbers are the confidence score from YOLOv5, and labels (marked with 'Insect(L)') indicate an annotated insect that was not detected by the model.

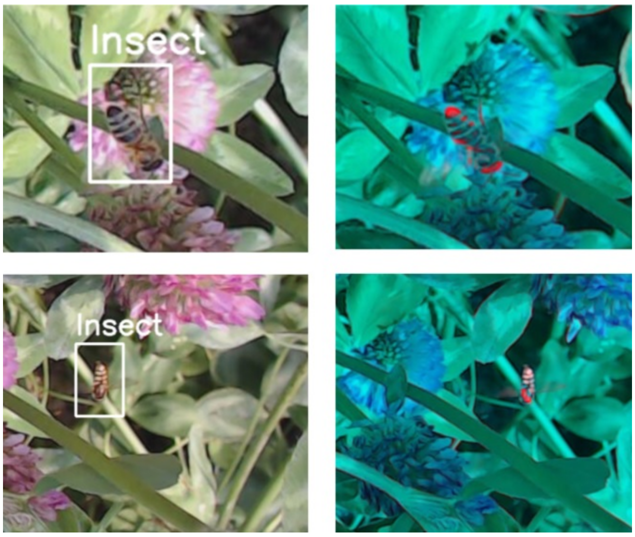

Figure S3. A honeybee *Amphis melifera* (top) and a hoverfly *Eupeodes corolla* (bottom) shown in RGB and motion-enhanced images.

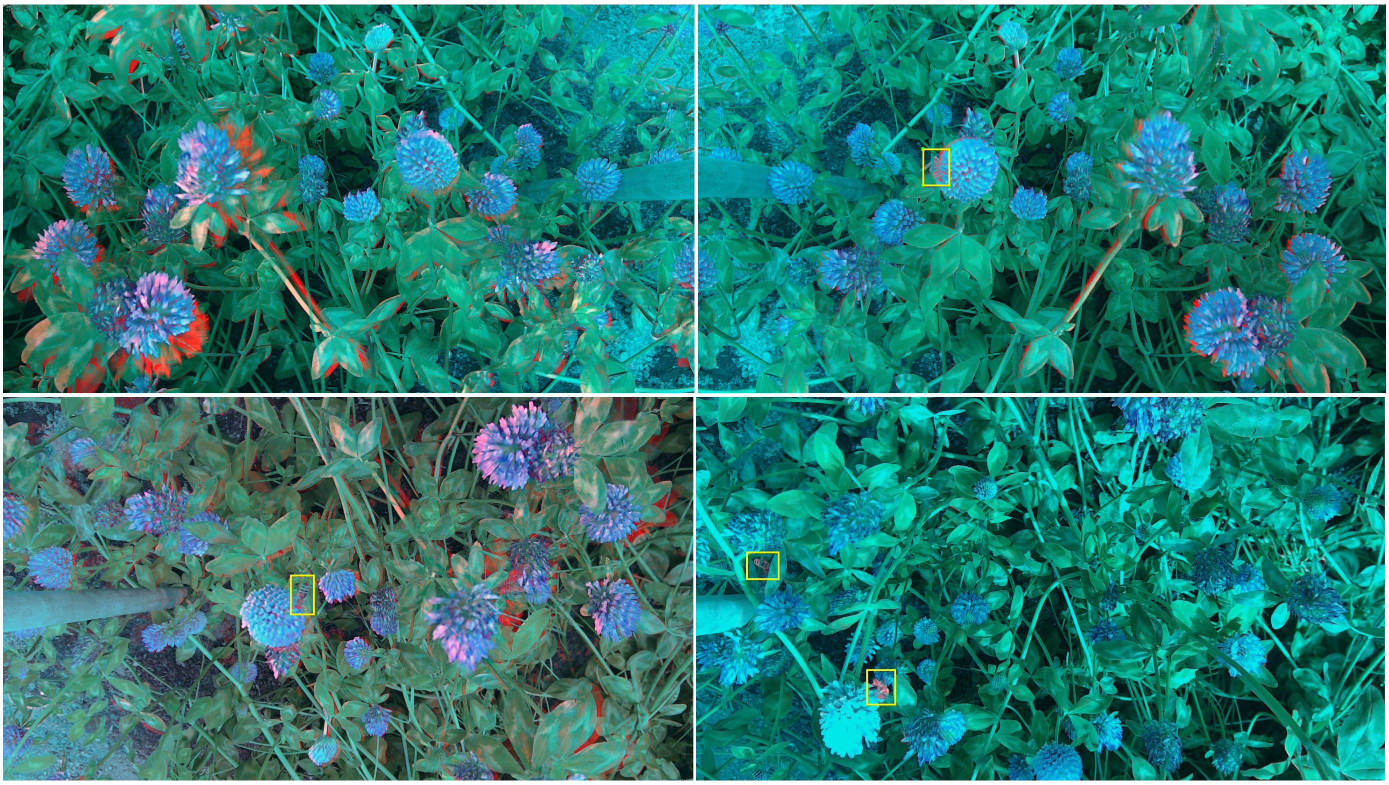

Figure S4. Motion-enhanced images that show how plants move between captured images with an interval of 30 seconds. Bees are marked with a yellow bounding box.

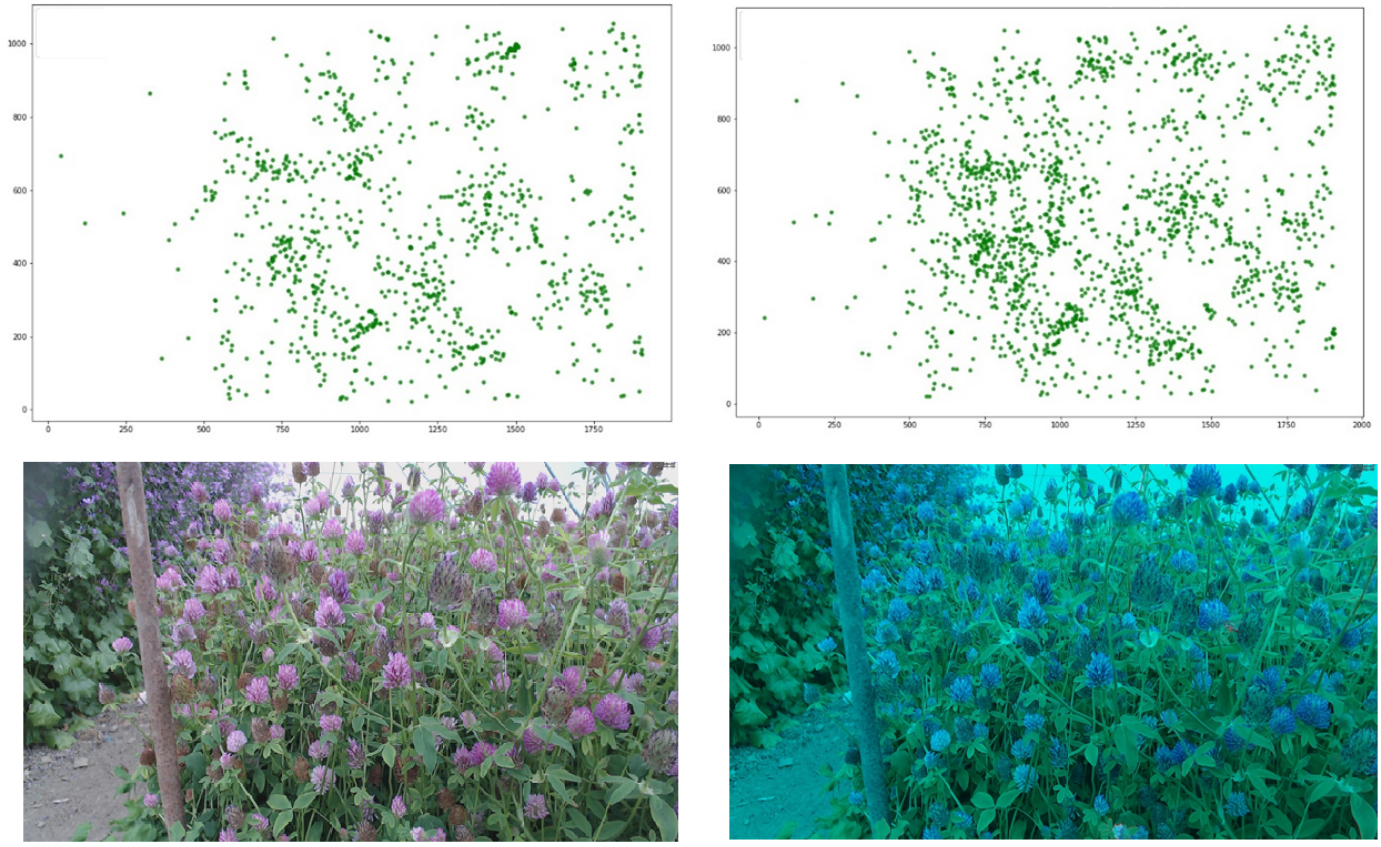

Figure S5. Motion-enhanced images show more detected insects. Green marks show the detections found in week 27 from camera S4-0. The YOLOv5 model is trained with RGB (left image with 1,072 detections) and motion-enhanced images (right image with 1,691 detections). Numbers on the axes are pixel coordinates.
